# Synthesis and In Vitro Metabolic Profiling of the Selective Androgen Receptor Modulator (SARM) LY305

**DOI:** 10.1002/rcm.10124

**Published:** 2025-08-15

**Authors:** Giorgi Kobidze, Tristan Möller, Hui‐Chung Wen, Francesco Paolo Busardò, Mario Thevis

**Affiliations:** ^1^ Department of Excellence‐Biomedical Sciences and Public Health Università Politecnica Delle Marche Ancona Italy; ^2^ Center for Preventive Doping Research, Institute of Biochemistry German Sport University Cologne Cologne Germany; ^3^ Faculty of Chemistry University of Cologne Cologne Germany

**Keywords:** doping, HPLC‐HRMS, LY305, phase‐I metabolites, phase‐II metabolites, SARM

## Abstract

**Rationale:**

2‐Chloro‐4‐[[(1R,2R)‐2‐hydroxy‐2‐methyl‐cyclopentyl]amino]‐3‐methyl‐benzonitrile (LY305) is a transdermal selective androgen receptor modulator (SARM) that has been under development as a potential therapeutic for conditions involving muscle wasting, osteoporosis, or hypogonadism. Due to its proven anabolic effects, its potential (and illicit) use in sport must be taken into consideration, necessitating information on its biotransformation for the implementation of adequate target analytes into routine doping control analytical procedures. In this study, the synthesis of LY305 and in vitro‐derived metabolites of the SARM are described.

**Methods:**

LY305 was synthesized by a Buchwald–Hartwig amination using 2‐chloro‐4‐iodo‐3‐methylbenzonitrile and (1*R*,2*R*)‐2‐amino‐1‐methyl‐cyclopentanol. The in vitro metabolism experiments were conducted using human liver microsomes (HLM) and the S9 fraction to allow for studying phase‐I and phase‐II biotransformations. For the detection and separation of LY305 and its metabolites, liquid chromatography–high‐resolution tandem mass spectrometry was used.

**Results:**

Overall, 18 metabolites were detected, nine of which were identified as phase‐I metabolites and an additional nine were attributed to phase‐II conjugates. Metabolic reactions were as follows: hydroxylation, dehydrogenation, oxidation, oxidation and hydroxylation, *O‐*glucuronidation, hydroxylation with subsequent glucuronidation, and bis‐hydroxylation with subsequent glucuronidation.

**Conclusions:**

The transdermal SARM LY305 was successfully synthesized and subjected to in vitro metabolic studies, yielding chromatographic and mass spectral data that support improving comprehensive anti‐doping tests. To the best of our knowledge, no published experimental data exist that report on the in vitro metabolic profile of LY305, a substance that might undergo further (pre)clinical evaluation.

## 1 Introduction

Selective Androgen Receptor Modulators (SARMs) have received considerable and growing attention in anti‐doping research, fueled by their structural diversity and associated analytical challenges, as well as the increasingly frequent reporting of adverse analytical findings in sports drug testing programs [1]. Similar to anabolic androgenic steroids, SARMs target the androgen receptor (AR), a member of the nuclear receptor superfamily. Members of this superfamily share structural similarities and play pivotal roles in metabolism, immune functions, reproduction, etc. [2]. The AR can be selectively activated by SARMs, making them drug candidates of particular interest, which are being developed to support medical therapies where promoting bone and muscle growth is required [3]. However, to this point, no SARM has yet received clinical approval by any government regulatory authority. Nevertheless, they have become available on poorly regulated online markets as supplement products and illegally marketed as “research chemicals” website suppliers but are clearly targeting human performance and image enhancement. SARMs have frequently been marketed as dietary supplements, available for consumers online, and investigations into such supplements demonstrated that product quality controls were questionable.

SARMs pose a considerable risk to the integrity of sports in both human and animal sports due to their potential performance‐enhancing properties. Consequently, SARMs are prohibited in sports, e.g., in human sports by the regulations of the World Anti‐Doping Agency (WADA), [4] Among the SARMs currently subject to anti‐doping research, 2‐chloro‐4‐[[(1R,2R)‐2‐hydroxy‐2‐methyl‐cyclopentyl]amino]‐3‐methyl‐benzonitrile (LY305) is specifically relevant. LY305 is being designed as the first transdermally bioavailable SARM and was developed to avoid some of the published side effects of oral SARMs, namely significant suppression of high‐density lipoprotein cholesterol (HDLc) and evidence demonstrated tolerability and safety after transdermal delivery in healthy volunteers in phase I clinical studies [5]. Due to its alternative route of administration, pharmacokinetics and metabolism of LY305 may differ from other previously investigated SARMs, a fact that might become particularly important in consideration of recent reports highlighting the possibility of inadvertent dermal drug exposure scenarios [6]. However, no published data currently exist on the metabolic fate of LY305 and, to the best of the authors knowledge, no adverse analytical finding has been reported to date concerning this SARM.

**FIGURE 1:**
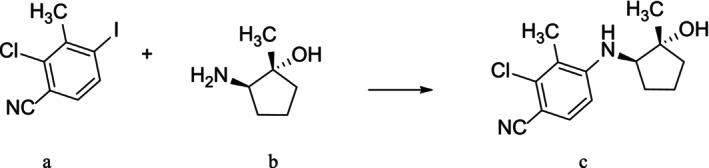
Synthetic route to produce LY305. Reaction conditions: Cs_2_CO_3_, 1,4‐Dioxane, Pd (dba)_2_, xantphos, 100°C, 24 h. a: 2‐Chloro‐4‐iodo‐3‐methylbenzonitrile, b: (1*R*,2*R*)‐2‐Amino‐1‐methyl‐cyclopentanol, c: LY305.

Therefore, the aim of this study was to synthesize the (transdermally bioavailable) non‐steroidal SARM LY305 (Figure 1) and to characterize its phase‐I and phase‐II in vitro‐derived metabolites using human liver microsomes (HLM) and liver S9 fraction. Thereby, critical data on potential metabolites that may be encountered in authentic doping control samples are generated, facilitating target analyte implementation into routine doping control methods employing liquid chromatography high‐resolution tandem mass spectrometry (LC‐HRMS).

## 2 Experimental

### 2.1 Reagents and Standards

2‐Chloro‐4‐iodo‐3‐methylbenzonitrile and (1*R*,2*R*)‐2‐amino‐1‐methyl‐cyclopentanol were purchased from BLD Pharmatech GmbH (Reinbek, Germany). Uridine diphosphate glucuronic acid (UDPGA), D‐saccharic acid‐1,4‐lactone (SL), 3′‐phosphoadenosine‐5′‐phosphosulfate (PAPS), bis (dibenzylidenacetone)palladium(0), xantphos, cesium carbonate (Cs_2_CO_3_), 1,4‐dioxane, and Celite were purchased from Sigma Aldrich (Darmstadt, Germany). Formic acid (FA), S9 fraction, and HLM were obtained from Thermo Scientific (Bremen, Germany). Dimethyl sulfoxide (DMSO) was obtained from Carl Roth (Kalsruhe, Germany). *β*‐Glucuronidase was acquired from Roche Diagnostics GmbH (Mannheim, Germany). Acetonitrile (ACN), ethyl acetate (EtOAc), and *n*‐pentane were purchased from VWR (Darmstadt, Germany). Nicotinamide adenine dinucleotide phosphate (NADPH)‐regenerating system was supplied by Promega (Madison, Wisconsin, USA). Methanol (MeOH) was purchased from J.T.Baker (Phillipsburg, NJ, USA). Ultrapure water was received from a Barnstead GenPure xCAD Plus from Thermo Scientific.

Column chromatography was performed using silica gel (63–200 μm) from Supelco (Sigma Aldrich). For reaction control and control of the cleaning step with column chromatography, thin layer chromatography (TLC) plates from Merck (Darmstadt, Germany) were used.

### 2.2 Instrumentation

#### 2.2.1 NMR Spectroscopy

Bruker Avance I 300 and Bruker Avance III 499 (Bremen, Germany) were used for recording NMR spectra. ^1^H NMR spectra were acquired at a frequency of 300.1 or 499.9 MHz, while ^13^C NMR spectra were acquired at a frequency of 125.7 MHz. Peak assignments were assisted with two‐dimensional spectra (H,H‐COSY, H,C‐HMBC, H,C‐HMQC). The chemical shift *σ* and the coupling constant ^3^J or ^4^J are indicated in ppm and in Hz, respectively. The multiplicity is classified as singlet (s), doublet (d), triplet (t), doublet of doublet (dd), triplet of triplet (tt) and multiplet (m).

#### 2.2.2 HPLC‐HRMS Instrumentation

A HPLC 1290 Infinity II coupled to a mass spectrometer (6546 Q‐TOF) equipped with a dual jet stream electrospray ionization source (Dual AJS ESI) (Agilent Technologies, Waldbronn, Germany) was used in the study. Data were acquired with MassHunter Workstation Quantitative Analysis 10.0 Software (Agilent Technologies). The separation of LY305 and its generated metabolites was achieved with an Accucore Biphenyl column (100 × 2.1 mm dimension and packed with 2.6 μm particles) from Thermo Scientific (Bremen, Germany). The total chromatographic run time was 25 min with a mobile phase composed of water with 0.1% FA (mobile phase A) and ACN with 0.1% FA (mobile phase B) at a flow rate of 0.25 mL/min. Gradient elution was applied to the method, starting with 2% of mobile phase B, which was maintained until 2 min; after 2 min, mobile phase B was gradually increased to 65% within 16 min, then within 1 min, mobile phase B was increased to 95% and maintained for 3 min; from 22.01 min, the chromatographic system was reequilibrated to the starting conditions for 3 min. Full scan acquisition mode in positive and negative ionization mode was used with a resolution of 60,000 full width at half maximum (FWHM) and a scan range of *m*/*z* 70–800. MS^2^ data for LY305 and its metabolites were acquired in negative ionization mode in a range of *m/z* 30–400, with an extraction window of 1.3 *m/z*. Autosampler oven temperature was set at 10°C. Collision energy was set to 25 eV. Optimal Dual AJS ESI conditions were as follows: capillary voltage 4000 V, source temperature 325°C, cone gas flow rate 10 L/min, desolvation gas flow rate 12 L/min.

### 2.3 In Vitro Metabolic Assay

HLM with a combination of liver S9 fraction was used to conduct in vitro metabolic reactions. For this, a 1 mg/mL solution of LY305 in DMSO was diluted into 50 mM phosphate buffer (pH 7.4), which contained 5 mM MgCl_2_ to produce a 200 μM LY305 working solution.

For the phase‐I metabolism, 10 μL of the working solution, 5 μL of the phosphate buffer, 25 μL of the NADPH generating system solution in phosphate buffer (prepared by mixing NADPH generating system solution A and solution B, with a ratio of 5:1, which was diluted in phosphate buffer 8.3 times), 5 μL of HLM (20 mg/mL), and 5 μL of the S9 (20 mg/mL) fraction were combined to yield a total volume of 50 μL, which was incubated at 37°C for 24 h.

For the phase‐II metabolites, an additional volume of 10 μL of SL (50 mM), 10 μL of UDPGA (25 mM), 10 μL of PAPS (20 μM) (working solutions of each were prepared in the phosphate buffer), 10 μL of phosphate buffer, 5 μL of HLM, and 5 μL of S9 fraction were added and incubated at 37°C for another 24 h. Negative control samples were prepared as well, one without HLM and S9 fraction and another one without LY305. For the control of the metabolic reaction, compound SARM 2f was incubated using the same approach. Metabolic reactions were quenched using ice‐cold ACN after 24 and 48 h for phase‐I and phase‐II biotransformations, respectively. Then the mixture was vortexed and centrifuged at 13,000 rpm for 5 min, and the supernatant was taken into a clean tube and evaporated under reduced pressure at 50°C for 1 h. After evaporation, the remaining residue was reconstituted into 100 μL of ACN:H_2_O (90/10, v/v).

## 3 Results

### 3.1 Synthesis of LY305

The synthesis of LY305 was described by Saeed et al. in 2015 [7], and while following the overall principle of this protocol, LY305 was synthesized in this study using a Buchwald–Hartwig amination with a catalyst system of bis‐(dibenzylidenacetone)‐palladium(0) (Pd (dba)_2_) and xantphos (see Figure 1) [8] For this, 2‐chloro‐4‐iodo‐3‐methylbenzonitrile (0.10 g; 0.87 mmol; 1 eq), (1*R*,2*R*)‐2‐amino‐1‐methyl‐cyclopentanol (0.26 g; 0.95 mmol; 1.09 eq) and Cs_2_CO_3_ (0.34 g; 1.04 mmol; 1.2 eq) were placed in a Schlenk tube under inert atmosphere and dissolved in 1,4‐dioxane (4 mL). After the addition of bis (dibenzylideneacetone)dipalladium (0.04 g; 0.04 mmol; 5 mol‐%) and xantphos (0.06 g; 0.10 mmol; 12 mol‐%), the mixture was stirred at 100°C for 24 h. The reaction was cooled to room temperature and filtered using Celite. The solvent was removed under reduced pressure. For purification, a column chromatography with silica gel (63–200 μm) as stationary phase was used. Multiple washing steps were conducted using different ratios of *n‐*pentane/EtOAc as mobile phase. Different fractions were collected in glass tubes, and the purity of the product was assessed using TLC and LC‐HRMS. Fractions containing exclusively LY305 were combined and evaporated under reduced pressure. The final product was isolated as a yellow solid with a yield of 0.15 g (59%).

^1^HNMR (CDCl3; 500 MHz): δ 1.26 (s; 3H), δ 1.44 (m, 1H), δ 1.74 (m, 6H), δ 2.22 (s, 3H), δ 6.82 (*d*, *J* = 8.7 Hz, 1H), δ 7.36 (*d*, *J* = 8.6 Hz, 1H) (^1^HNMR data shown in the Figure S1).

^13^CNMR (CDCl3; 125 MHz): δ 13.67 ppm (CH3), δ 19.90 ppm (CH2), δ 23.50 ppm (CH3), δ 32.10 ppm (CH2), δ 40.18 ppm (CH2), δ 63.37 ppm (CH), δ 80.78 ppm (Cquat.), δ 100.10 ppm (Cquat.), δ 109.11 ppm (CH), δ 118.30 ppm (Cquat.), δ 119.68 ppm (Cquat.), δ 132.80 ppm (CH), δ 136.35 ppm (Cquat.), δ 150.34 ppm (Cquat.) (^13^CNMR data shown in the Figure S2).

### 3.2 ESI/collision‐Induced Dissociation of LY305

**FIGURE 2:**
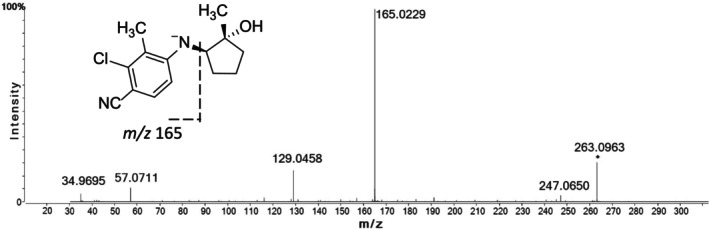
High‐resolution product ion mass spectrum obtained for LY305 after negative electrospray ionization.

LY305 was detected in both positive and negative ionization mode. The response of the compound (and its metabolites) in negative electrospray ionization was found to be superior; hence, negative ionization was chosen for all further investigations. Using the analytical method described above, LY305 eluted at 14.4 min with [M‐H]^−^ at *m*/*z* 263.0963 (2.2 ppm) featuring an experimentally determined elemental composition of C_14_H_16_ClN_2_O^−^. At the collision energy of 25 eV, a characteristic product ion (assigned to deprotonated 4‐amino‐2‐chloro‐3‐methylbenzonitrile) was observed at *m/z* 165.0229 (2.4 ppm), attributable to the elimination of cyclopentan‐1‐ol and a subsequent loss of HCl, leading to the product ion at *m/z* 129.0458 (3.0 ppm) Further minor product ions were found at *m/z* 57.0711 and 34.9695, which plausibly match the tentatively assigned species of deprotonated butane (1.7 ppm) and a chlorine ion (2.8 ppm) (Figure 2).

### 3.3 Metabolite Identification

**FIGURE 3:**
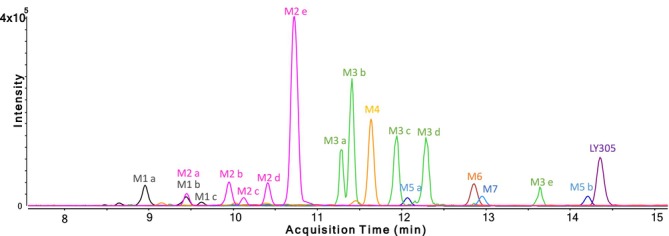
Extracted ion chromatogram of LY305 and its biotransformation products following 24 h of incubation with human liver microsomes and S9 fraction. Mass tolerance 5 ppm. Color legend: gray: bis‐hydroxylation + glucuronidation (M1), pink: hydroxylation + glucuronidation (M2), green: hydroxylation (M3), golden: *O*‐glucuronidation (M4), light blue: dehydrogenation (M5), orange: oxidation (M6), dark blue: oxidation + hydroxylation (M7).

**FIGURE 4:**
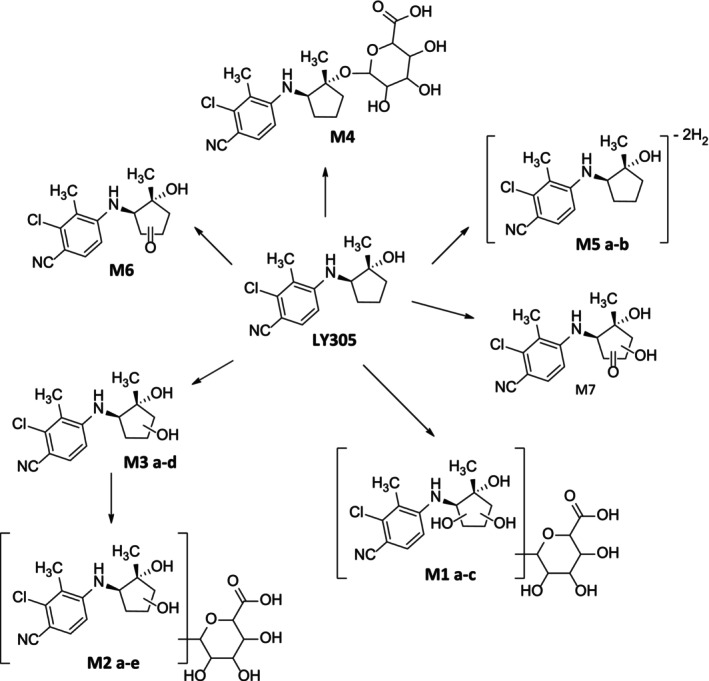
Summary of suggested metabolic transformations of LY305 as observed in human in vitro experiments.

An integrated non‐targeted and targeted analysis approach for identification of in vitro‐generated metabolites of LY305 was used. Overall, 18 potential metabolites (named M1 a–c, M2 a–e, M3 a–e, M4, M5 a,b, M6 and M7, based on the ascending order of the chromatographic elution depicted in Figure 3) were identified using in vitro incubation experiments. From these 18 metabolites, nine were identified as possible phase‐I metabolites, and an additional nine were identified as possible phase‐II conjugates. Phase‐I metabolites were most likely produced through the following metabolic reactions: hydroxylation (M3 a–e), dehydrogenation (M5 a and M5 b), oxidation (M6) and oxidation with additional hydroxylation (M7). Phase‐II metabolites were produced through: glucuronidation (M4), hydroxylation and subsequent glucuronidation (M2 a–e) and bis‐hydroxylation followed by glucuronidation (M1 a–c). The suggested human in vitro metabolic pattern of LY305 is summarized in Figure 4.

#### 3.3.1 Mass Spectrometric Characterization of Phase‐I Metabolites

**FIGURE 5:**
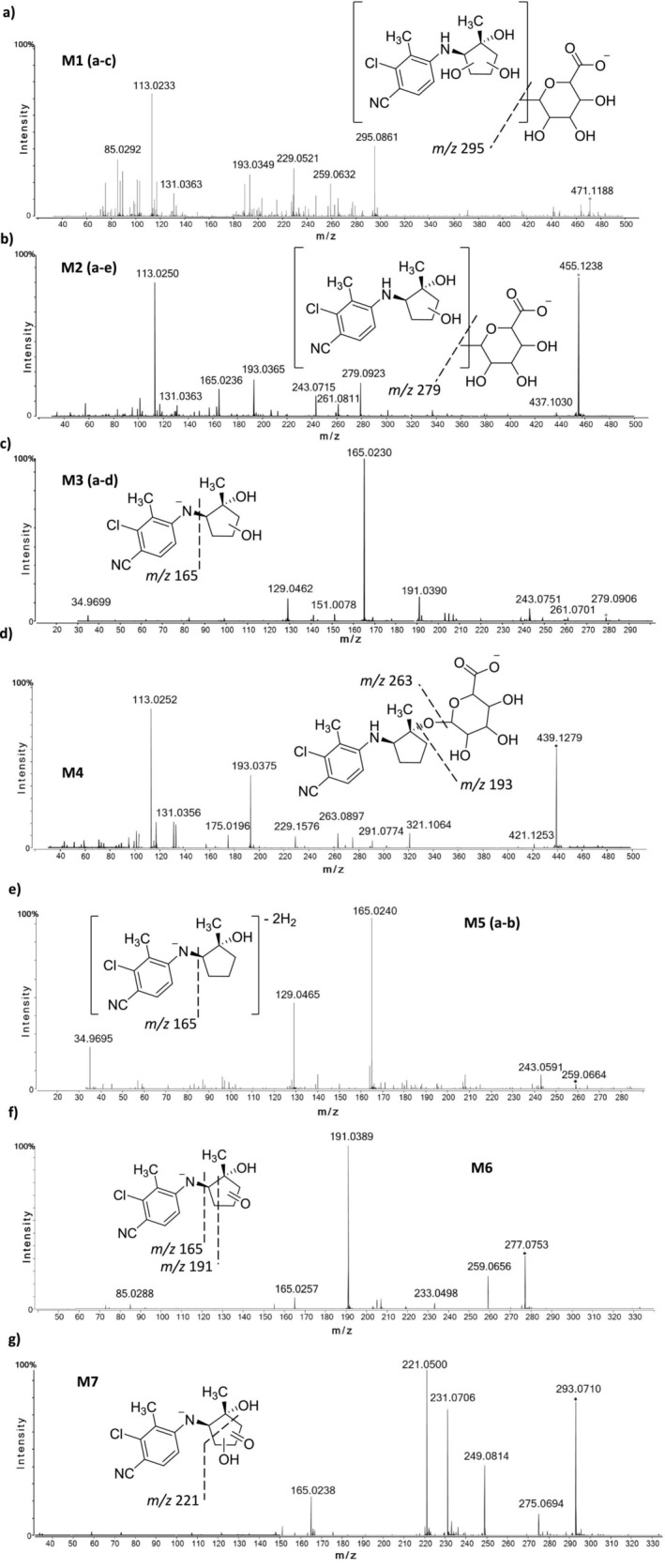
High‐resolution product ion mass spectra of deprotonated LY305 metabolites and suggested dissociations.

Overall, 9 phase‐I metabolites were identified after 24 h of HLM incubation of LY305. Hydroxylated metabolites (M3 a–e) were most abundant among all phase‐I metabolites. For this group of biotransformation products, which exhibited a deprotonated molecule at *m/z* 279.0906, a total of five distinct peaks were detected. Here, the four signals with the retention time (RT) of 11.3, 11.4, 11.9, and 12.4 min yielded similar MS^2^ spectra, indicating the presence of stereo‐ or regioisomers. At a collision energy of 25 eV, the product ion mass spectra of these analytes presented an intense signal at *m/z* 165.0230 (3.0 ppm) with an elemental composition of C_8_H_6_ClN_2_^−^. The co‐existence of other product ions at *m/z* 191.0390 (4.7 ppm) with an elemental composition of C_10_H_8_ClN_2_^−^ and *m/z* 129.0462 (3.0 ppm) with an elemental composition of C_8_H_5_N_2_^−^ as well as signals at *m/z* 261.0701 and *m/z* 243.0751 that were suggested to represent ions produced by sequential water elimination reactions (18 Da) corroborate the formation of mono‐hydroxylated metabolites, bearing the hydroxyl group at the cyclopentane moiety (Figure 5c). Conversely, the signal at RT = 13.6 min yielded a MS^2^ spectrum (MS^2^ data shown in Figure S3) exhibiting a peak at *m/z* 180.0097 (0.5 ppm) with an elemental composition of C_8_H_5_ClN_2_O^**−**^, suggesting the hydroxylation of LY305 at the benzyl moiety. A second signal at *m/z* 261.0826 was assigned to the loss of water from the deprotonated molecule. For this metabolite (M3 e), sequential dehydration was not observed, which further substantiated the proposal of aromatic ring hydroxylation [9].

M5 a and M5 b eluted at 12.0 and 14.3 min, respectively. Those metabolites appear to be the product of dehydrogenation of LY305, with an elemental composition C_14_H_12_ClN_2_O^−^, as suggested by the 4.0293 Da mass difference compared to the unmodified LY305. The abundant product ion at *m/z* 165.0228 (1.8 ppm) and the (subsequent) cleavage of HCl generating the second most intense product ion at *m/z* 129.0462 (0 ppm) indicated that the 4‐amino‐2‐chloro‐3‐methylbenzonitrile moiety remained unmetabolized (Figure 5e).

The M6 metabolite eluted at 12.9 min, with an elemental composition C_14_H_14_ClN_2_O_2_^−^, as suggested by the +13.9792 Da shift from LY305, yielding a precursor ion at *m/z* 277.0753 (1.4 ppm). The most abundant product ion observed at *m/z* 191.0389 (4.1 ppm) with an elemental composition of C_10_H_8_ClN_2_^−^ was assigned to the deprotonated 2‐chloro‐3‐methyl‐4‐(vinylamino)‐benzonitrile. Considering also minor product ions at *m/z* 259.0656 (4.6 ppm) and 165.0229 (2.4 ppm), assigned to the loss of water (−18 Da) and the deprotonated species of 4‐amino‐2‐chloro‐3‐methylbenzonitrile, respectively, suggested the formation of an oxygenated analog of LY305 (Figure 5f).

The M7 metabolite eluted at 13.0 min, with an elemental composition C_14_H_14_ClN_2_O_3_^−^, deduced from a + 29.9741 Da shift compared to the accurate mass of LY305. The deprotonated molecule was detected at *m/z* 293.0710 (4.0 ppm) and the product ion mass spectrum's base peak was at *m/z* 221.0500 under the chosen MS/MS conditions. This product ion was tentatively postulated to result from a cyclopentane ring cleavage, retaining an N‐linked oxopropyl residue at the 4‐amino‐2‐chloro‐3‐methylbenzonitrile moiety, with an elemental composition of C_11_H_10_ClN_2_O^−^. For the ion at *m/z* 231.0706, an elemental composition of C_12_H_8_ClN_2_O^−^ was determined, and other minor signals at *m/z* 249.0814 and *m/z* 275.0694 were assigned to deprotonated 4‐(2‐hydroxycyclopentylamino)‐2‐chloro‐3‐methylbenzonitrile and water loss from the M7 metabolite, respectively (Figure 5g).

#### 3.3.2 Mass Spectrometric Characterization of Phase‐Ii Metabolites

Overall, 9 Phase‐II metabolites were detected, all of which presented a glucuronic acid conjugation. Attributed metabolic reactions included *O*‐glucuronidation, hydroxylation + glucuronidation, and bis‐hydroxylation + glucuronidation.

M4 eluted at 11.6 min, with a +176.0322 Da shift from the unmodified substrate LY305, yielding a deprotonated molecule at *m/z* 439.1279 (0 ppm) and thus suggesting glucuronidation. Since enzymatic hydrolysis of the conjugate using *β*‐glucuronidase resulted in a complete cleavage of the conjugate, *O*‐ rather than *N*‐glucuronidation of LY305 was proposed. The most abundant product ions (*m/z* 175.0196, 131.0356, and 113.0252 [mass error < 5 ppm]) have previously been assigned and identified as characteristic markers of glucuronic acid residues [10], and other diagnostic product ions were suggested to represent the deprotonated LY305 and a water loss from M4 (Figure 5d).

Metabolites M2 a–e featured a deprotonated molecule at *m/z* 455.1238 (2.4 ppm) and eluted at 9.5, 9.9, 10.1, 10.4, and 10.7 min, with similar MS^2^ spectra. With a mass shift of +192.0281 Da compared to the intact substrate, a combination of reduction (+2H), hydroxylation (+O), and glucuronidation was proposed. Similar to M4, the breakdown of the glucuronide residue produced the most abundant product ions for this metabolite as well at *m/z* 113.0250 and 131.0363. Ions representing deprotonated LY305 and hydroxylated LY305 were also detected in the MS^2^ experiment at *m/z* 165.0236 and 279.0923 (Figure 5b).

M1 a–c eluted at 9.0, 9.4, and 9.6 min with intact and deprotonated molecular ions at *m/z* 471.1188 (2.5 ppm), translating to an elemental composition of C_20_H_24_ClN_2_O_9_^−^. The overall mass difference of +208.0231 Da compared to LY305 suggested a combination of bis‐hydroxylation and glucuronidation. Ions typical for glucuronide dissociation products prevailed also here, and additional characteristic ions matched the deprotonated bis‐hydroxy‐LY305 and its fragment ions produced by the losses of water molecules at *m/z* 295.0861 (4.0 ppm) and 259.0632 (4.6 ppm), respectively (Figure 5a). A summary of analyte details is provided in Table 1.

**TABLE 1:** Proposed elemental compositions and biotransformations, retention times, and accurate molecular masses of LY305 and its phase‐I and phase‐II metabolites after 24 h of incubation in negative electrospray ionization mode. Mass tolerance 5 ppm.

ID	Metabolic reaction	Monoisotopic mass (Da)	Precursor ion [M‐H]^−^ (*m/z*) theoretical	Precursor ion [M‐H]^−^ (*m/z*) experimental	Elemental composition	Most intense product ions [M‐H]^−^ (*m/z*)	Retention time (min)
M1 a M1 b M1 c	Bis‐hydroxylation + Glucuronidation	472.1249	471.1176	471.1188	C_20_H_25_ClN_2_O_9_	113.0233 295.0861	9.0 9.4 9.6
M2 a M2 b M2 c M2 d M2 e	Hydroxylation + Glucuronidation	456.1299	455.1227	455.1238	C_20_H_25_ClN_2_O_8_	113.0250 279.0923	9.5 9.9 10.1 10.4 10.7
M3 a M3 b M3 c M3 d M3 e	Hydroxylation	280.0979	279.0906	279.0906	C_14_H_17_ClN_2_O_2_	165.0230 129.0462	11.3 11.4 11.9 12.4 13.6
M4	*O*‐Glucuronidation	440.1350	439.1278	439.1279	C_20_H_25_ClN_2_O_7_	113.0252 193.0375	11.6
M5 a M5 b	Dehydrogenation	260.7188	259.0644	259.0664	C_14_H_13_ClN_2_O	165.0240 129.0465	12.0 14.3
M6	Oxidation	278.0822	277.0749	277.0753	C_14_H_15_ClN_2_O_2_	191.0389 259.0656	12.9
M7	Oxidation + Hydroxylation	294.0771	293.0698	293.0710	C_14_H_15_ClN_2_O_3_	221.0500 231.0706	13.0
LY305		264.1029	263.0957	263.0963	C_14_H_17_ClN_2_O	165.0229 129.0458	14.4

## 4 Discussion and Conclusion

In this study, the synthesis and in vitro metabolism of the (transdermal) SARM LY305 were conducted. To date, limited data on the metabolism of this compound, in vitro or in vivo, were published, and considering the substance's relevance especially in anti‐doping, these findings are critical to ensure that this new doping threat is characterized and laboratories are able to include LY305 and/or its characteristic metabolites in the analysis of SARMs for sports drug testing programs. In this in vitro study, main metabolic reactions were assigned to hydroxylation and glucuronidation of the SARM, which suggests considering LY305 glucuronide (C_20_H_25_ClN_2_O_7_ with diagnostic precursor‐product ion pairs of *m/z* 439—*m/z* 175, *m/z* 439—*m/z* 131, and *m/z* 439—*m/z* 113) and hydroxylated LY305 glucuronide (C_20_H_25_ClN_2_O_8_, with diagnostic precursor‐product ion pairs of *m/z* 455—*m/z* 193, *m/z* 455—*m/z* 165, and *m/z* 455—*m/z* 113) in non‐hydrolyzed urine samples for sports drug testing purposes. In case of enzymatic or chemical cleavage of phase‐II metabolites during sample preparation, LY305 (C_14_H_17_ClN_2_O, diagnostic precursor‐product ion pairs of *m/z* 263—*m/z* 165, *m/z* 263—*m/z* 247, and *m/z* 263—*m/z* 129) and hydroxylated LY305 (C_14_H_17_ClN_2_O_2_, diagnostic precursor‐product ion pairs of *m/z* 279—*m/z* 165, *m/z* 279—*m/z* 191, and *m/z* 279—*m/z* 129) are recommended as markers to document LY305 consumption.

The results obtained in this study are consistent with the literature data by Krishnan et al., where *O*‐glucuronidation and mono‐oxidation at the cyclopentane ring were mentioned as a main biotransformation in the human body [5]. However, the metabolic profile of LY305 may differ when applied transdermally since this route of administration circumvents hepatic first‐pass effects and, furthermore, skin‐specific metabolic reactions might impact the overall pattern of biotransformation products as reported for other anabolic agents [6, 11, 12]. Nevertheless, the hepatic metabolism is expected to considerably influence the formation of (potential) target analytes for routine doping controls concerning LY305. Approaches towards identifying the individual impact of organs on the metabolism of drug candidates are manifold, with static as well as dynamic models (e.g., organs‐on‐a‐chip) providing valuable insights [13, 14, 15, 16] However, future in vivo studies contributing to understanding the pharmacokinetic properties of LY305 and its metabolites after transdermal application (including humans) appear to be critical for sports drug testing purposes, as highlighted recently by the transdermal delivery of ostarine through a contaminated neoprene hamstring sleeve, which resulted in an adverse analytical finding [17] The transdermal SARM LY305 represents a new and unique threat to sport, and the chemical synthesis and in vitro identification of relevant metabolites is an important first step, but more directed research on this substance would help to ensure that any use as a doping agent will be detectable.

## Author Contributions

**Giorgi Kobidze:** conceptualization, methodology, formal analysis, investigation, writing – original draft, writing – review and editing. **Tristan Möller:** conceptualization, methodology, formal analysis, investigation, writing – review and editing. **Hui‐Chung Wen:** formal analysis, investigation. **Francesco Paolo Busardò:** methodology, writing – review and editing. **Mario Thevis:** conceptualization, methodology, resources, formal analysis, investigation, supervision, validation, writing – review and editing.

## Conflicts of Interest

The authors declare no conflicts of interest.

## Supporting information


**Figure S1:** rcm10124‐sup‐0001‐Suppl_info.docx. ^1^H‐NMR‐Spectra of LY305.
**Figure S2:**: ^13^C‐NMR‐Spectra of LY305.
**Figure S3:**: ESI‐MS^2^ spectrum of M3 e.

## Data Availability

The data that support the findings of this study are available from the corresponding author upon reasonable request.
