# Supplementary material for: Synthesis and In Vitro Metabolic Profiling of the Selective Androgen Receptor Modulator (SARM) LY305
Source: Rapid Commun Mass Spectrom. 2025 Aug 15;39(22):e10124. doi: 10.1002/rcm.10124 (PMC12355332; doi:10.1002/rcm.10124)
Supplement: Supplementary file 1 — Figure S1: rcm10124‐sup‐0001‐Suppl_info.docx. 1H‐NMR‐Spectra of LY305. Figure S2:: 13C‐NMR‐Spectra of LY305. Figure S3:: ESI‐MS2 spectrum of M3 e. [file RCM-39-e10124-s001.docx]

**Supporting information**

**Synthesis and in-vitro metabolic profile of the selective androgen receptor modulator (SARM) LY305**

Giorgi Kobidze^1,2^, Tristan Möller^2^, Hui-Chung Wen^3^, Francesco Paolo Busardò^1^, Mario Thevis^2^*

^1^Department of Excellence-Biomedical Sciences and Public Health, Università Politecnica delle Marche, 60121 Ancona, Italy

^2^Center for Preventive Doping Research, Institute of Biochemistry, German Sport University Cologne, Am Sportpark Müngersdorf 6, 50933 Cologne, Germany

^3^Faculty of Chemistry, University of Cologne, Greinstraße 4-6, 50939 Cologne, Germany

Corresponding author: Mario Thevis, e-mail: thevis@dshs-koeln.de


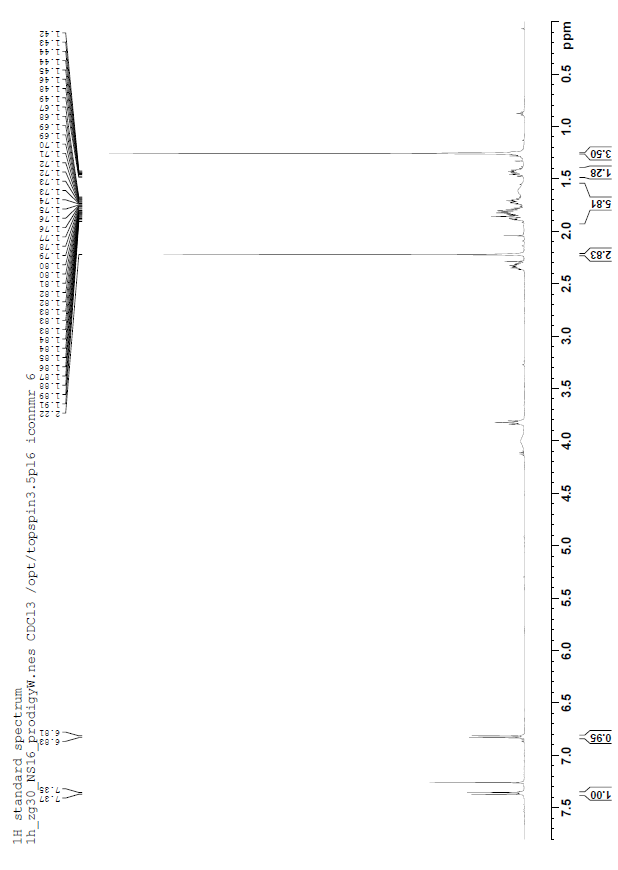


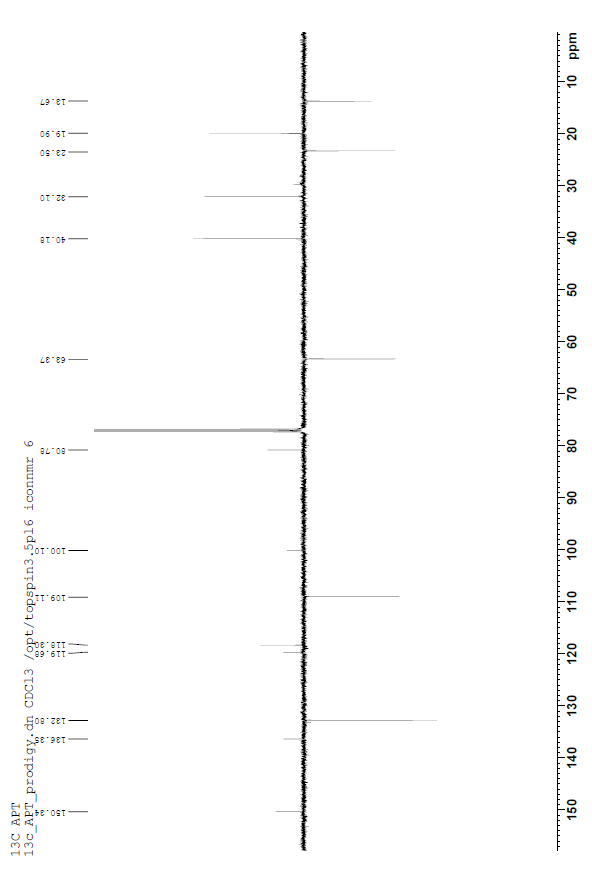


**Figure 1**: ^1^H-NMR-Spectra of LY305

**Figure 2**: ^13^C-NMR-Spectra of LY305


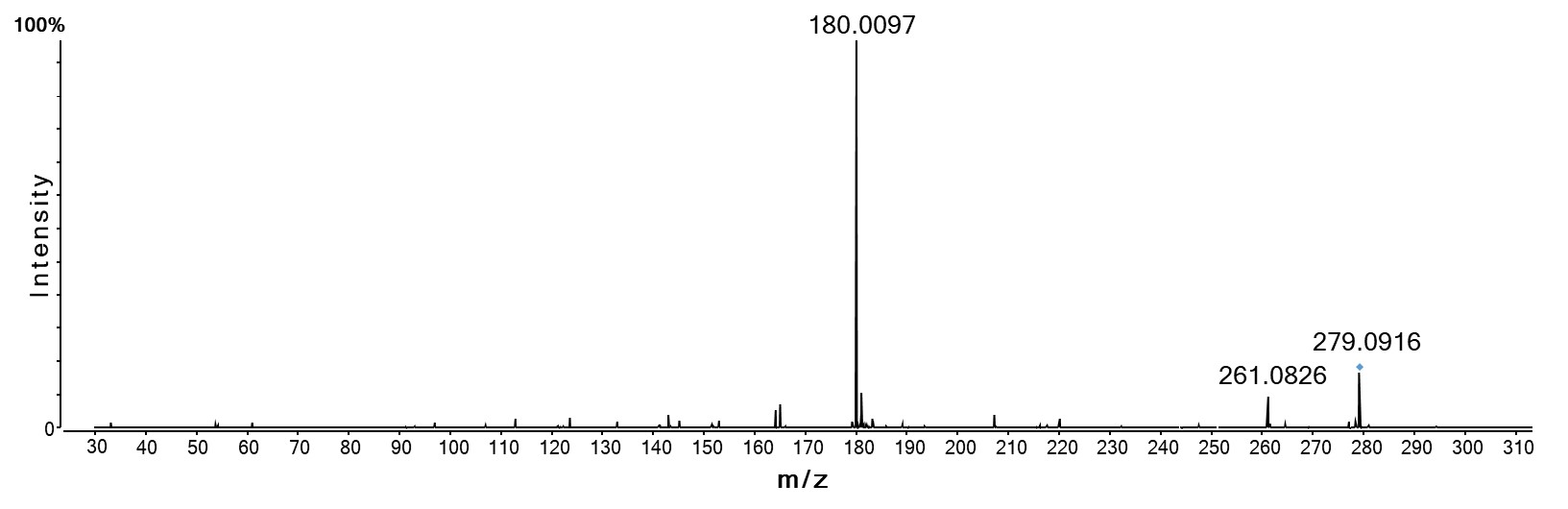


**Figure 3**: ESI-MS^2^ spectrum of M3 e
